# Isolated Radial Head Dislocation in Children: A Case Series Exploring Diagnosis, Treatment Approaches, and Long-Term Outcomes

**DOI:** 10.7759/cureus.81659

**Published:** 2025-04-03

**Authors:** Ismail Benomar, Kenza Bendeddouhce, Loubna Aqqaoui, Houda Oubejja, Fouad Ettayebi

**Affiliations:** 1 Pediatric Surgery, Children's Hospital of Rabat, Faculty of Medicine and Pharmacy, Mohammed V University, Rabat, MAR

**Keywords:** children, closed reduction, dislocation, isolated, radial head

## Abstract

Isolated radial head dislocation in children is a rare but significant injury, often resulting from trauma. It is clinically challenging to diagnose due to its subtle symptoms and can lead to serious long-term consequences if not treated promptly. Early detection and treatment are crucial to prevent persistent elbow pain, stiffness, instability, and functional impairment.

This report details four cases of isolated radial head dislocation in children, each following trauma. All patients underwent closed reduction and were immobilized in a splint for three weeks. The cases were reviewed to highlight the importance of timely intervention and the outcomes of the treatment approach.

Despite advances in treatment techniques, the exact mechanism behind isolated radial head dislocation remains unclear. Radial head dislocations are more commonly seen in adults, and their presentation in children can be easily overlooked due to less pronounced clinical and radiological symptoms. A precise diagnosis is essential to avoid long-term complications such as instability and functional limitations.

Early diagnosis and appropriate treatment of isolated radial head dislocation in children are essential for preventing lasting effects. Further research is needed to understand the underlying mechanisms and optimize post-reduction management strategies to improve long-term outcomes.

## Introduction

Radial head dislocation in children is commonly associated with Monteggia fractures [1]. Isolated dislocation of the radial head, a rare condition, can be linked to congenital conditions such as trisomy 8 or Ehlers-Danlos syndrome [2]. Although more common in adults, post-traumatic radial head dislocation can be challenging to detect in children [3].

Chronic elbow discomfort, stiffness, and reduced elbow mobility can result from this injury if it is not recognized or treated appropriately, requiring more involved procedures. Prompt and effective reduction of the radial head is crucial for maintaining elbow joint mobility and integrity [3]. It is an exceptional entity, and only a few cases are reported in the literature [4].

In the absence of a concomitant ulnar fracture, traumatic radial head dislocation frequently goes undiagnosed [5]. It is still unclear exactly how isolated radial head dislocation occurs [6].

We present four distinct cases of radial head dislocation after trauma.

## Case presentation

Case 1

**Figure 1:**
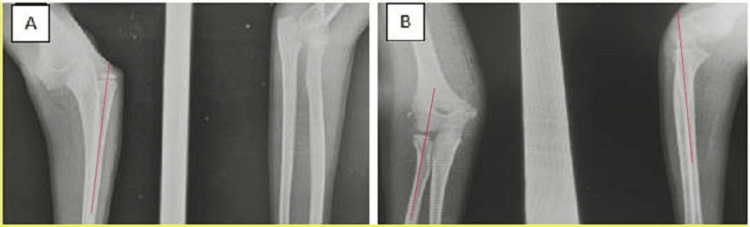
X-ray showing isolated dislocation of the radial head (A) and good alignment of the radiocapitellar line after closed reduction (B) A: The radiocapitellar line in red does not pass through the capitellum. B: After closed reduction, the radiocapitellar line passes through the capitellum.

After suffering a left elbow injury during a sporting event, a 10-year-old boy with no prior medical history visited the emergency room. The patient arrived with severe discomfort, elbow deformity, and complete functional impotence of the left upper limb. Elbow flexion and extension were impossible with a 20° range of motion, and passive pronation and supination were limited at 0°. Vascular and neurological evaluations were normal. The radial head was dislocated, but there was no ulnar injury visible on the initial radiographs. By applying pressure on the radial head, mild supination, and simple traction along the radius axis, closed reduction was accomplished. The success of the reduction was verified by a post-reduction X-ray. Although there was some minor residual edema, active range of motion was reestablished. A splint was applied for 21 days for immobilization to guarantee the stability of the reduction and lower the chance of relaxation. The patient was examined at 30 days, with complete recovery of limb mobility. At six months, there were no complications such as stiffness, instability, or pain (Figure 1).

Case 2

**Figure 2:**
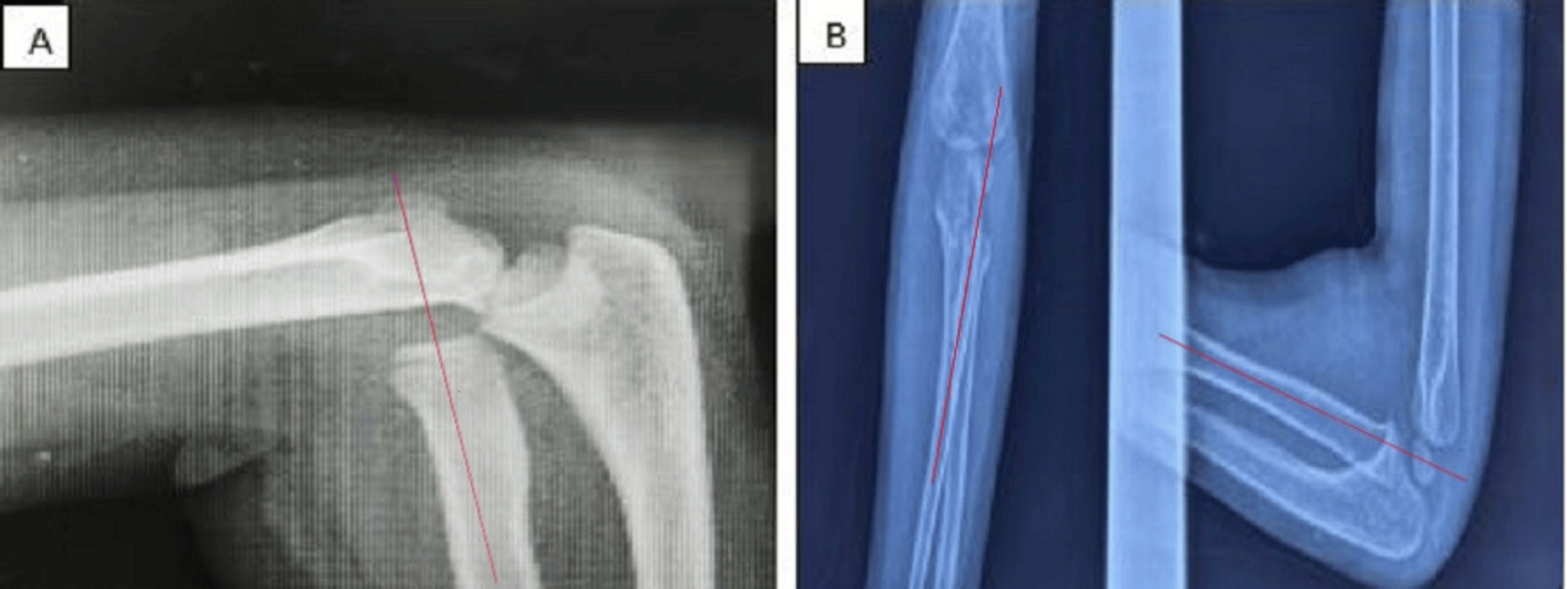
X-ray showing isolated dislocation of the radial head (A) and good alignment of the radiocapitellar line after closed reduction (B) A: The radiocapitellar line in red does not pass through the capitellum. B: After closed reduction, the radiocapitellar line passes through the capitellum.

After falling from a height, a six-year-old child with no prior medical history arrived at the emergency room with functional impotence of the right upper limb. Upon examination, there was limited prono-supination at 0° and excruciating elbow flexion and extension with a 30° range of motion. The nervous-vascular evaluation came out normal. An isolated lateral dislocation of the radial head was visible on the first radiographs, there was no ulnar injury visible, and closed reduction was likewise successful in reducing it. A post-reduction X-ray verified the success of the reduction. For three weeks, a palmar brachio-antebrachial splint was then used. The patient was examined at 30 days, with complete recovery of limb mobility, and at six months, there were no complications such as stiffness, instability, or pain (Figure 2).

Case 3

**Figure 3:**
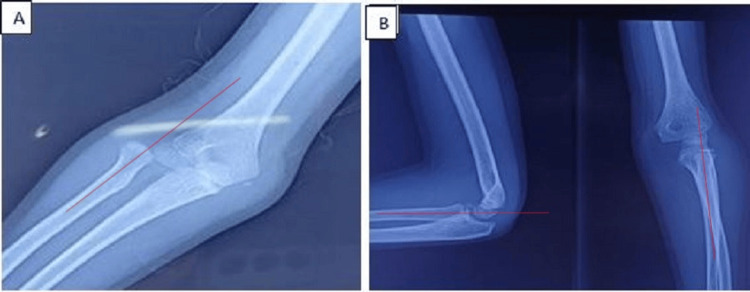
X-ray showing isolated dislocation of the radial head (A) and good alignment of the radiocapitellar line after closed reduction (B) A: The radiocapitellar line in red does not pass through the capitellum. B: After closed reduction, the radiocapitellar line passes through the capitellum.

A nine-year-old boy who had no prior history of any specific injuries arrived at the emergency department after falling off his bicycle. The patient arrived with severe discomfort, elbow deformity, and complete functional impotence of the left upper limb. Elbow flexion and extension were impossible with a 25° range of motion, and passive pronation and supination were limited at 0°. The radial head was dislocated, but there was no ulnar injury visible on the initial radiographs. A firm reduction with minor traction and supination was conducted. A post-reduction X-ray verified the success of the reduction. For three weeks, a palmar brachio-antebrachial splint was used. The patient was examined at 30 days, with complete recovery of limb mobility, and at six months, there were no complications such as stiffness, instability, or pain (Figure 3).

Case 4

**Figure 4:**
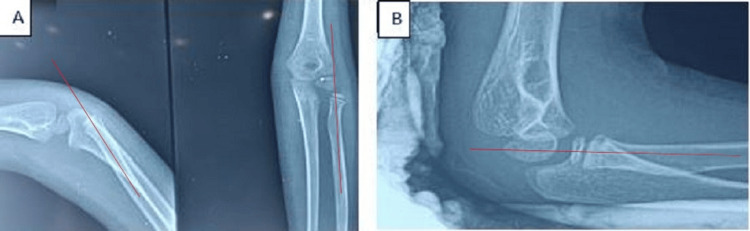
X-ray showing isolated dislocation of the radial head (A) and good alignment of the radiocapitellar line after closed reduction (B) A: The radiocapitellar line in red does not pass through the capitellum. B: After closed reduction, the radiocapitellar line passes through the capitellum.

A six-year-old boy with no specific history arrived at the emergency room after falling from a height. The patient arrived with severe discomfort, elbow deformity, and complete functional impotence of the left upper limb. Elbow flexion and extension were impossible with a 20° range of motion, and passive pronation and supination were limited at 0°. The radial head was dislocated (Figure 4A), but there was no ulnar injury. After undergoing a closed orthopedic reduction, he spent three weeks immobilized in a splint. The patient was examined at 30 days, with complete recovery of limb mobility, and at six months, there were no complications such as stiffness, instability, or pain (Figure 4).

## Discussion

Due to sometimes ambiguous history, inconclusive clinical signs, and relatively faint radiological markers, it is easy to overlook the diagnosis of radial head dislocation. The diagnosis is determined by a thorough radiographic examination [7].

Remote trauma-induced isolated radial head dislocations without an obvious ulnar lesion, such as Monteggia fracture, have been confused with congenital radial head dislocations [7]. Specific criteria for congenital radial head dislocation have been established by McFarland [8]. It is essential to pay attention to radiological indicators that point to a congenital rather than traumatic dislocation or subluxation, such as hypoplasia of the capitellum, a prominent ulnar epicondyle, a dome-shaped radial head, an elongated radial neck, and often a relatively short ulna (in relation to the radius) [9].

Dislocations of the radial head are most frequently related to sports activities and falling on an outstretched hand; however, the precise mechanism for injury is unclear [7].

Diagnosis can be easily misled or delayed. The radial head's spontaneous reduction and subsequent redislocation were held responsible for the delayed diagnosis [3].

Correct history taking, an exhaustive physical examination, and plain radiography films are essential for the precise identification of acute radial head dislocation using the radiocapitellar line. Patients usually complain of elbow pain and reluctance to move it after a fall. These patients frequently have a restricted range of motion and elbow edema, although physical examinations at this young age can occasionally not be practicable [3].

The supination with flexion technique is frequently employed to reduce radial head dislocation; a click signifies that the reduction into the annular ligament was successful [10].

Simple patient arm placement during imaging can occasionally result in reduction. However, the necessary technique depends on the precise mechanism of injury and the dislocation pattern [3].

There is controversy over the ideal forearm rotational position for radial head stability. Instead of immobilizing the forearm in neutral rotation or pronation, some authors have suggested immobilizing it in supination. Other authors have suggested neutral rotation following the manual reduction of the radial head [6].

The mechanism of injury might vary from a brief distraction of the radiocapitellar joint during severe pronation to a partial tear of the annular ligament. Acute traumatic radial head dislocations generally indicate an annular ligament injury from a direct elbow injury [10]. The nature of ligament injury influences the direction of dislocation: a ligament lesion of the annular ligament causes lateral dislocation, a lesion of the quadrate ligament causes posterolateral dislocation, and a lesion of the interosseous membrane causes anterior dislocation [6].

Chronic radial head dislocation can cause the elbow to become more valgus, which can then result in ulnar or radial nerve dysfunction. It can also limit flexion because of radial head obstruction, which causes stiffness and instability, which can lead to function loss [7].

Additionally, untreated or inadequately controlled radial head dislocation has been associated with post-traumatic osteoarthritis or elbow valgus deformity, which can limit flexion and have a long-term negative impact on upper limb function, as demonstrated by recent studies [7].

Large deformed radial heads, narrow radial necks, and ulnar bowing were common dysplastic alterations seen in misdiagnosed radial head dislocation. Longer delays between the time of injury and the reduction period were observed for more severe abnormalities [11].

There are numerous challenges in treating missing or chronic dislocations. Four weeks after the initial damage, there is very little chance of attaining a closed reduction; to restore normal anatomy, an open reduction is typically necessary. The surgical result for chronic radial head dislocation may be influenced by the patient's age and the interval between the damage and the start of treatment. When to execute the surgical reduction for the greatest outcome is the crucial question [12].

Surgery is required if there is irreducible reduction or when the dislocation fails to be resolved with non-surgical procedures. Although surgical methods differ from case to case, they typically involve ligament repairs or joint fixation procedures to stabilize the elbow and prevent deformity. Although they are somewhat uncommon, these approaches are crucial for preserving joint function in children who do not respond to conventional therapies [7].

Potential obstacles to closed reduction, such as the biceps tendon, joint capsule, brachialis muscle, and/or annular ligament, were highlighted by Shelton et al. [1] in their report of an isolated medial head dislocation that required open reduction since the coronoid blocked the closed reduction route [13].

## Conclusions

In summary, despite being rare in children, isolated radial head dislocations pose a serious clinical problem. To avoid problems and guarantee a full recovery, prompt discovery, suitable treatment with closed reduction, and appropriate immobilization are crucial.

Uncertainties remain regardless of improvements in the treatment of isolated radial head dislocation. More research is needed to figure out the precise mechanisms causing this dislocation, as well as optimal practices for post-reduction immobilization. To guarantee the best possible functional recovery and avoid long-term problems, future studies should concentrate on assessing long-term immobilization strategies and streamlining rehabilitation processes.
